# Increased risk of everolimus-associated acute kidney injury in cancer patients with impaired kidney function

**DOI:** 10.1186/1471-2407-14-906

**Published:** 2014-12-03

**Authors:** Sung Hae Ha, Ji Hyeon Park, Hye Ryoun Jang, Wooseong Huh, Ho-Yeong Lim, Yoon-Goo Kim, Dae Joong Kim, Ha Young Oh, Jung Eun Lee

**Affiliations:** Division of Nephrology, Department of Medicine, Samsung Medical Center, Sungkyunkwan University School of Medicine, Seoul, Korea; Departments of Internal Medicine, Dongincheon Gil Hospital, School of Medicine, Gachon University, Incheon, Korea; Division of Oncology, Departments of Medicine, Samsung Medical Center, Sungkyunkwan University School of Medicine, Seoul, Korea

**Keywords:** Everolimus, mTOR inhibitor, Adverse effect, Renal cell carcinoma, Acute kidney injury

## Abstract

**Background:**

Everolimus was recently introduced as a second-line treatment for renal cell carcinoma (RCC) and many other cancers. Several prospective studies have shown that serum creatinine levels are increased in a significant proportion of patients receiving everolimus. However, data on the occurrence of acute kidney injury (AKI) during everolimus treatment in clinical practice are sparse. Here, we report the incidence, risk factors, and clinical significance of AKI associated with everolimus treatment in patients with cancer.

**Methods:**

We analyzed patients who received everolimus for more than 4 weeks as an anticancer therapy. AKI was defined as increase in creatinine levels from baseline levels greater than 1.5-fold.

**Results:**

The majority of the 110 patients enrolled in this analysis had RCC (N=93, 84.5%). AKI developed in 21 (23%) RCC patients; none of the patients (N=17) with other cancers had AKI. Fourteen of 21 cases were considered to be everolimus-associated AKI, in which there were no other nephrotoxic insults other than everolimus at the onset of AKI. The incidence of AKI increased progressively as baseline estimated glomerular filtration rate (eGFR) decreased (10% in subjects with eGFR >90 mL/min/1.73 m^2^, 17% in subjects with eGFR 60–90 mL/min/1.73 m^2^, 28% in subjects with eGFR 30–60 mL/min/1.73 m^2^, and 100% in subjects with eGFR 15–30 mL/min/1.73 m^2^; *P*=0.029 for trend). Baseline eGFR was an independent risk factor for the development of everolimus-associated AKI (hazard ratio per 10 mL/min/1.73 m^2^ increase, 0.70; 95% confidential interval, 049–1.00; *P*=0.047). Nine of 14 patients with everolimus-associated AKI continued receiving the drug at a reduced dose or after a short-term off period. Administration of the drug was discontinued in four of 14 patients because of progression of an underlying malignancy. Only one patient stopped taking the drug because of AKI.

**Conclusions:**

This paper suggests that AKI is a common adverse effect of everolimus treatment, especially in subjects with impaired renal function. However, the occurrence of AKI did not require the discontinuation of the drug, and the treatment decision should be made via a multidisciplinary approach, including the assessment of the oncological benefits of everolimus and other therapeutic options.

## Background

Everolimus is a major active metabolite of sirolimus, which acts as a selective inhibitor of the mammalian target of rapamycin (mTOR) [1]. Everolimus has been developed as an immunosuppressant that is administered after solid organ transplantation based on its antiproliferative properties [1, 2]. In particular, an mTOR-inhibitor-based regimen in which calcineurin inhibitors are withdrawn or reduced has been evaluated as a maintenance immunosuppressant therapy to minimize calcineurin-inhibitor toxicity [2, 3]. Although mTOR inhibitors have been considered to lack nephrotoxicity when used alone, the combination of mTOR inhibitors and full-dose calcineurin inhibitors has been shown to exacerbate nephrotoxicity. Moreover, the nephrotoxicity of mTOR inhibitors has been demonstrated in patients with glomerulonephritis and in experimental animal models of glomerular injury [4, 5]. Recently, everolimus received approval for use in the treatment of advanced renal cell carcinoma (RCC) and several other cancers at a dose of 10 mg once daily, which is a higher dose than that used for immunosuppression [6–8]. Increased serum creatinine level was one of the frequently reported laboratory abnormalities during observed in a phase 3 trial of everolimus for metastatic renal cell cancer [6]. However, the information regarding the nephrotoxicity associated with everolimus, especially in cancer patients with clinical settings, is sparse. Therefore, we conducted this research to evaluate the incidence, severity, risk factors, and prognosis of acute kidney injury (AKI) in patients receiving everolimus as an anticancer therapy. We were particularly interested in patients with RCC who already had a decreased mass of functioning nephrons because of nephrectomy, invasion of cancer, or previous treatment with vascular endothelial growth factor receptor/tyrosine kinase inhibitors (VEGFR-TKIs).

## Methods

### Patients

Between January 2009 and September 2013, 140 adult patients (>18 years of age) who took everolimus as an anticancer treatment at the Samsung Medical Center were identified using electronic databases. We excluded patients who received everolimus for less than 4 weeks (N = 12), for whom there were insufficient data (N = 17), or for whom the baseline estimated glomerular filtration rate (eGFR) was less than 15 mL/min/1.73 m^2^ (N = 1). Data from 110 patients were analyzed. At the Samsung Medical Center, advanced RCC or hepatocellular carcinoma (HCC) that failed VEGFR-TKI treatment was an indication for everolimus treatment. Generally, patients received 10 mg of everolimus once daily; however, the dose and schedule could be modified according to toxicity and tolerability. Most patients were followed every 4 weeks and laboratory tests including creatinine were performed at every visit. This research was approved by the Institutional Review Board of the Samsung Medical Center.

### Data collection

Demographic data including age, sex, body mass index, the malignancy that was targeted by everolimus, past medical history of diabetes mellitus, hypertension, nephrectomy, medication with angiotensin converting enzyme (ACE) inhibitors, angiotensin receptor blockers (ARBs) and diuretics, and prior treatment with VEGFR-TKIs were extracted from electronic medical records. Hypertension was defined as a systolic blood pressure >140 mmHg, a diastolic blood pressure >90 mmHg, or self-reported hypertension with or without ongoing pharmacological treatment. Diabetes mellitus was defined as a history of type 1 or type 2 diabetes mellitus treated pharmacologically or controlled by diet. Information regarding total dosage, treatment duration, and reason for discontinuation of everolimus, as well as dose modification after AKI, was also collected. Laboratory data, including baseline serum creatinine level (defined as the latest creatinine within 2 months before treatment), eGFR, and urinalysis were extracted. eGFR was calculated using the Chronic Kidney Disease Epidemiology Collaboration (CKD-EPI) equation as follows: eGFR = 141 × minimum (creatinine/κ, 1)^α^ × maximum (creatinine/κ, 1)^-1.209^ × 0.993^age^ × 1.018 (if female), where κ is 0.7 for women and 0.9 for men and α is -0.329 for women and -0.411 for men [9]. Serum creatinine levels were determined every 4 weeks during treatment, and 1, 3, and 6 months after the discontinuation of everolimus.

The primary outcome was the development of AKI, which was defined according to the Acute Dialysis Quality Initiative (ADQI) criteria. Briefly, patients were classified in the “risk” category if serum creatinine increased 1.5-fold or eGFR decreased >25%, in the “injury” category if serum creatinine increased 2-fold or eGFR decreased >50%, and in the “failure” category if serum creatinine increased 3-fold or eGFR decreased >75% [10]. Time to AKI was defined as the interval between the start of everolimus therapy and the onset of AKI. AKI category was determined based on peak serum creatinine. Recovery from AKI was defined as the return to a serum creatinine within 1.2-fold of the baseline value. Everolimus-associated AKI was defined as cases in which there were no other nephrotoxic insults at the onset of AKI, such as nephrotoxic drugs, contrast media, hypotension, infection, urinary tract obstruction, or volume depletion.

### Statistical analysis

Data are expressed as the median with interquartile range (IQR), or absolute number with percentages. Intergroup differences were compared using the Mann–Whitney *U* test for continuous variables and Fisher’s exact test or chi-squared analysis for categorical variables. The cumulative incidence of AKI was determined using the Kaplan–Meier method. Uni- and multivariate Cox proportional models were fitted to identify risk factors of AKI. The multivariate analysis included variables with a *P*-value < 0.1 according to the univariate analysis. We regarded *P*-values < 0.05 as significant. All statistical analyses were conducted using SPSS 21.0 (IBM Inc., Armonk, NY).

## Results

### Baseline characteristics of the subjects according to underlying malignancy

A total of 110 patients met the inclusion criteria, and the majority of patients (N = 93) received everolimus to treat RCC. The remaining patients had HCC (N = 7), pancreas neuroendocrine tumors (N = 5), lymphoma (N = 2), or other tumors (melanoma, leiomyosarcoma, and rectal carcinoid, N = 1 for each). Baseline characteristics are shown for the two groups of patients according to underlying malignancy, RCC vs non-RCC, as these two groups showed quite different baseline characteristics (Table 1). The median age was 59 years (range, 52–67 years) in the RCC group and 54 years (48–59 years) in the non-RCC group. In the RCC group, the median eGFR was 63 mL/min/1.73 m^2^ (51–76 mL/min/1.73 m^2^) and 83 patients (89%) had decreased renal function (defined as GFR values <90 mL/min/1.73 m^2^), whereas most individuals in the non-RCC group had normal renal function. In the RCC group, all patients received VEGFR-TKI therapy, and 74 (80%) patients received radical nephrectomy before treatment with everolimus. Twenty-five (28%) patients in the RCC group had proteinuria at the baseline. The total dosage and duration of everolimus treatment were 1155 mg (670–1900 mg) and 20 weeks (12–36 weeks), respectively, in the RCC group.

**Table 1:** **Baseline characteristics of the subjects according to underlying malignancy**

	RCC (N = 93)	Non-RCC (N = 17)
Male sex , no. (%)	77 (82%)	10 (63%)
Age (years)	59 (52, 67)	54 (48, 59)
BMI (kg/m^2^)	23.5 (20.0, 25.2)	23.8 (21.4, 25.3)
Diabetes mellitus (%)	17 (18%)	0 (0%)
Hypertension (%)	33 (36%)	2 (11%)
ACE inhibitor/ARB (%)	15 (16%)	1 (6%)
Diuretics (%)	40 (43%)	5 (29%)
Previous TKI Treatment (%)	93 (100%)	9 (53%)
Radical nephrectomy	74 (80%)	0 (0%)
Creatinine (mg/dL)	1.19 (1.01, 1.43)	0.78 (0.64, 0.86)
eGFR (mL/min/1.73 m^2^)	63 (51, 76)	104 (94, 132)
>90	10 (11%)	14 (82%)
60–90	41 (44%)	2 (12%)
30–60	40 (43%)	1 (6%)
15–30	2 (2%)	0 (0%)
Proteinuria (%)	25 (28.1%)	0 (0%)
Everolimus		
Total dose (mg)	1155 (670, 1900)	790 (332, 1825)
Duration (weeks)	20 (12, 36)	12 (6, 26)

Data are presented as the median (IQR) or number (%).

RCC, renal cell carcinoma; BMI, body mass index; ACE inhibitor, angiotensin converting enzyme inhibitor; ARB, angiotensin receptor blocker; TKI, tyrosine kinase inhibitor; eGFR, estimated glomerular filtration rate.

### Cumulative incidence of AKI

**Figure 1:**
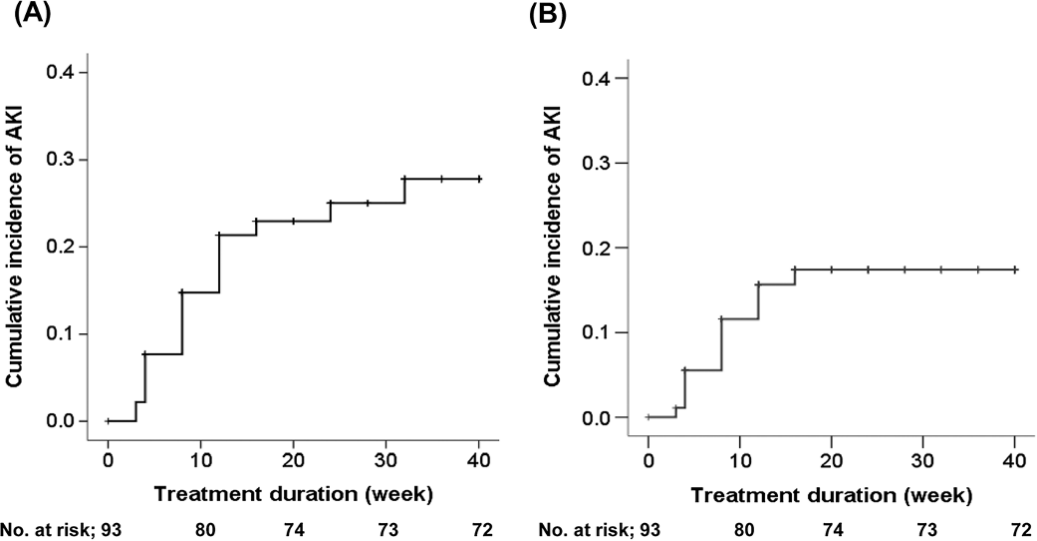
**Cumulative incidence of all-cause AKI (A) and everolimus-associated AKI (B) in the RCC group.**

AKI developed in 21 (23%) patients in the RCC group during everolimus treatment, whereas none of the patients in the non-RCC group experienced AKI. After exclusion of the patients with other nephrotoxic insults at the onset of AKI, everolimus-associated AKI developed in 14 (16.2%) patients. Figure 1A presents the cumulative incidence of AKI in the RCC group. Most cases of AKI (N = 19, 90%) occurred within 16 weeks of everolimus treatment, and all everolimus-associated AKI cases occurred within 16 weeks of treatment, with a median interval of 8 weeks (4–12 weeks) (Figure 1B).

### Association between AKI risk and baseline eGFR in the RCC group

**Figure 2:**
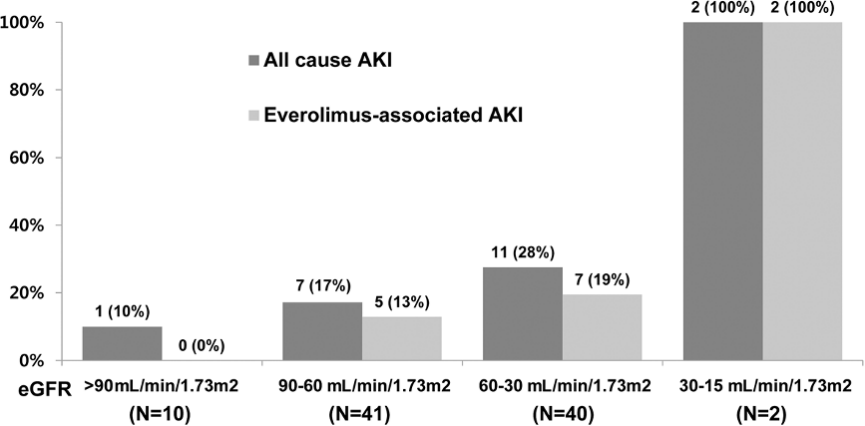
**Incidence of AKI according to baseline eGFR categories in the RCC group.** The incidence of all-cause AKI and everolimus-associated AKI increased progressively with decreasing eGFR (*P =* 0.029 and *P =* 0.004 for trend, respectively).

The incidence of all-cause AKI increased progressively as the baseline eGFR decreased. Figure 2 shows that the incidence of AKI was 10% in patients with a baseline eGFR >90 mL/min/1.73 m^2^, 17% in those with a baseline eGFR of 60–90 mL/min/1.73 m^2^, 28% in those with a baseline eGFR of 30–60 mL/min/1.73 m^2^, and 100% in those with a baseline eGFR of 15–30 mL/min/1.73 m^2^ (*P =* 0.029 for trend). The incidence of everolimus-associated AKI also increased progressively with decreasing eGFR (*P =* 0.004 for trend). All patients with a baseline eGFR <30 mL/min/1.73 m^2^ experienced AKI.

To identify the risk factors of AKI in patients with RCC, we used Cox proportional hazard models (Table 2). On univariate analyses, older age and lower baseline eGFR were associated with a higher risk of everolimus-associated AKI. Multivariate analysis revealed that baseline eGFR was the only risk factor for AKI, and that an eGFR increase of 10 mL/min/1.73 m^2^ was associated with a 0.7-fold lower risk of AKI (95% confidence interval, 0.49–1.00; *P =* 0.047). The presence of proteinuria at the baseline was not independently associated with a higher risk of AKI.

**Table 2:** **Risk factors of everolimus-associated AKI in the RCC group: Cox proportional hazard models**

Variable	Univariate analysis		Multivariate analysis*	
	HR (95% CI)	***P***-value	HR (95% CI)	***P***-value
Age (per 10 years)	1.83 (1.05–3.21)	0.034	1.54 (0.84–2.81)	0.162
Male (vs female)	0.85 (0.19–3.82)	0.836		
DM	0.70 (0.16–3.14)	0.643		
HTN	0.74 (0.23–2.36)	0.609		
eGFR (per 10 mL/min/1.73 m^2^)	0.74 (0.57–0.96)	0.022	0.70 (0.49–1.00)	0.047
Radical nephrectomy	0.64 (0.20–2.06)	0.456		
Proteinuria	2.23 (0.75–6.63)	0.150		
ACE inhibitor/ARB	1.21 (0.34–4.34)	0.769		
Diuretics	1.09 (0.38–3.13)	0.880		

AKI, acute kidney injury; RCC, renal cell carcinoma; HR, hazard ratio; CI, confidence interval; eGFR, estimated glomerular filtration rate; ACE inhibitor, angiotensin converting enzyme inhibitor; ARB, angiotensin receptor blocker.

*This model includes age, sex, and eGFR.

### Outcome and clinical significance of everolimus-associated AKI in the RCC group

Next, we examined the outcome of everolimus-associated AKI (N = 14) according to the ADQI criteria. Ten patients who remained in the AKI-risk category continued everolimus treatment without renal deterioration. In half of these patients, the dosage was reduced to 5 mg per day, and two patients held off the medication for 1 week and 1 month, respectively, and resumed treatment without dose modification. The remaining three patients discontinued the drug because of progression of the underlying cancer. In three patients in the AKI-injury category, kidney function was recovered after the discontinuation or the reduction of the dosage of the drug; one of these three patients withheld medication for 2 weeks, and resumed the therapy at 50% of the previous everolimus dose, whereas another patient took everolimus with a 50% dose reduction without discontinuation of the medication. The remaining patient in the AKI-injury category discontinued everolimus because of the development of pneumonia, and not of AKI, and kidney function recovered to the baseline level. One patient in the AKI-failure category discontinued everolimus treatment eventually, and kidney function recovered thereafter.

To assess the effects of AKI on treatment decision in RCC patients, we compared treatment duration and reason for drug discontinuation between the AKI and non-AKI groups (Table 3). Treatment duration was 18 weeks (9–35 weeks) and 20 weeks (12–36 weeks) in the AKI and non-AKI groups, respectively, and the total dose of everolimus was 1050 mg (615–1913 mg) and 1172 mg (683–1915 mg) in the AKI and non-AKI groups, respectively (not significant; NS). The most common reason for drug discontinuation in both groups was progression of an underlying malignancy (67% in the AKI group and 68% in the non-AKI group; NS).

**Table 3:** **Treatment duration and reason for final cessation of everolimus treatment in the RCC group**

	AKI group	Non-AKI group	***P***-value
Treatment duration (weeks)	18 (9, 35)	20 (12, 36)	NS
Total dose (mg)	1050 (615, 1913)	1173 (683, 1915)	NS
Discontinuation of drug	15 (71%)	53 (74%)	NS
Reason for discontinuation			NS
Disease progression	10 (67%)	35 (66%)	
Adverse effect	2 (13%)	9 (17%)	
Self-withdrawal	3 (20%)	9 (17%)	

RCC, renal cell carcinoma; AKI, acute kidney injury; NS, not significant.

## Discussion

This retrospective analysis examined the incidence, risk factors, and clinical implications of the development of AKI during everolimus treatment in real-world cancer patients. Twenty-three percent of patients who received everolimus to treat RCC experienced AKI, and 67% of AKI events were considered everolimus-associated AKI without other nephrotoxic insults. In contrast to RCC patients, AKI events were not observed in any of the patients with other cancers, for whom baseline eGFR was much higher than the levels detected in patients with RCC. Baseline eGFR was the only independent risk factor of everolimus-associated AKI among patients with RCC. Differences in treatment duration and in the reason for drug discontinuation were not observed between the AKI group and non-AKI groups, which indicates that the occurrence of AKI did not have a high impact on therapeutic decision making by clinicians.

As many new chemotherapeutic agents have emerged in recent decades, nephrologists should be alerted to the potential nephrotoxicity of new drugs [11–14]. VEGFR-TKI is a representative target agent with well-established nephrotoxicity [11, 15]. Everolimus is familiar to nephrologists as an alternative immunosuppressant to calcineurin inhibitors after kidney transplantation, with the advantage of lack of nephrotoxicity [1–3]. However, everolimus has been examined as a treatment for various cancers and renal adverse effects have been reported [6, 16]. In fact, the incidence and severity were divergent in clinical studies and target cancers, which confused clinicians regarding the recognition of drug nephrotoxicity [17–19]. This research clearly showed that AKI was not uncommon in subjects with impaired kidney function, but was rare in subjects with normal kidney function. Impaired kidney function at the baseline is a general feature in subjects with RCC who have started to take everolimus. Therefore, clinicians should be cautious about potential nephrotoxicity when prescribing everolimus to RCC patients.

It is not surprising that most RCC patients have decreased kidney function when they initiate everolimus treatment. Currently, everolimus is indicated in metastatic RCC after progression on VEGFR-TKI therapy [20, 21]. Thus, patients have a high probability of reduced functioning in nephrons because of previous radical nephrectomy, the presence of a neoplastic mass replacing renal parenchyma, or previous exposure to VEGFR-TKI therapy. We assumed that nephrons with reduced function render RCC patients vulnerable to the adverse renal effects of everolimus. Several studies have demonstrated that mTOR inhibitors have nephrotoxicity in injured kidneys [5, 22]. The combination of mTOR inhibitors with full-dose calcineurin inhibitors exacerbates the nephrotoxicity of the drug [1]. In addition, everolimus treatment converts the reversible glomerulonephritis into chronic progressive disease in *Thy1* models via the inhibition of glomerular repair [5]. Moreover, everolimus treatment induces renal deterioration and proteinuria in the remnant kidney model [22]. Consistently, baseline eGFR was an independent risk factor of everolimus-associated AKI in this analysis.

The observation that the nephrotoxicity of everolimus was evident in patients with RCC compared with kidney transplant recipients who also had reduced nephron functioning was not an unexpected finding. The dosage of everolimus as an anticancer treatment is 10 mg per day, which is about three times higher than that used for immunosuppression in transplantation patients [1, 2, 6, 7]. An experimental study showed that everolimus-induced glomerular injury developed in a dose-dependent manner [5].

It is noteworthy that everolimus treatment was continued or resumed in most patients with AKI without renal deterioration, unless there was progressive disease or other significant adverse events. Most of the AKI events (13 of 14) were mild and nonprogressive (categorized into AKI-risk or AKI-injury according to ADQI criteria) during everolimus treatment for 20 weeks. This finding has important clinical significance when considering that the drug is indicated for patients with few therapeutic options. In addition, decreased renal function was recovered after the cessation of treatment, including one patient who was in the AKI-failure category.

There were some limitations to this research. First, as a retrospective analysis, selection and misclassification biases were inevitable. However, everolimus treatment in cancer patients is standardized at our center, and serum creatinine is monitored regularly, which might minimize those biases. Second, the effects of everolimus-associated AKI on patient mortality were not elucidated in this research. Third, we did not evaluate the incidence of proteinuria and increment of preexisting proteinuria, which are other renal adverse effects of anti-angiogenic drugs. In addition, this research did not provide any information on the histological features of everolimus-associated AKI, because none of subjects who experienced AKI underwent kidney biopsy, probably because the AKI was mostly mild and reversible.

## Conclusions

In conclusion, we demonstrated that AKI associated with everolimus, which is used as an anticancer therapy, is not uncommon in subjects with impaired kidney function, whereas it is rare in subjects with normal kidney function. Therefore, clinicians should be cautious about potential nephrotoxicity when prescribing everolimus to patients with decreased kidney function, in whom serial measurements of serum creatinine are needed. In addition, everolimus treatment could be continued at a reduced dose or after a short-term off period even in patients with AKI without renal deterioration. Therefore, the treatment decision should be made using a multidisciplinary approach that includes the assessment of the oncological benefit of everolimus and other therapeutic options for cancer in each individual. A large-scale, prospective study is needed to clarify the incidence of everolimus-associated AKI and its impact on patients’ survival.

## Abbreviations

mTOR: Mammalian target of rapamycin
RCC: Renal cell carcinoma
AKI: Acute kidney injury
VEGFR-TKIs: Vascular endothelial growth factor receptor/tyrosine kinase inhibitors
eGFR: Estimated glomerular filtration rate
HCC: Hepatocellular carcinoma
ACE inhibitor: Angiotensin converting enzyme
ARB: Angiotensin receptor blocker
CKD-EPI: Chronic Kidney Disease Epidemiology Collaboration
ADQI: Acute Dialysis Quality Initiative
IQR: Interquartile range.
